# Waste Materials as a Resource for Production of CMC Superabsorbent Hydrogel for Sustainable Agriculture

**DOI:** 10.3390/polym13234115

**Published:** 2021-11-26

**Authors:** Vojkan Miljković, Ivana Gajić, Ljubiša Nikolić

**Affiliations:** Faculty of Technology, University of Niš, Bulevar Oslobođenja 124, 16000 Leskovac, Serbia; ivananikolic1991@gmail.com (I.G.); nljubisa@gmail.com (L.N.)

**Keywords:** waste materials, carboxymethyl cellulose, superabsorbent hydrogel, agriculture

## Abstract

Waste materials are receiving more attention as concerns about the future of our planet increase. Cellulose is the most common substance in agricultural waste. Agricultural wastes containing cellulose are misplaced resources that could be reused in various fields for both environmental and economic benefits. In this work, 32 different kinds of waste are investigated for chemical modification in order to obtain carboxymethyl cellulose for the production of a superabsorbent hydrogel that can be applied in agriculture. A brief literature review is provided to help researchers wishing to obtain carboxymethyl cellulose by carboxymethylation starting with waste materials. We also provide details about methods to obtain as well as verify carboxymethylation. Carboxymethyl cellulose (CMC), as a constituent of cellulosic water and superabsorbent hydrogels with applications in agriculture, is described. Superabsorbent hydrogels with CMC are able to absorb huge amounts of water and are biodegradable.

## 1. Introduction

Agricultural waste material is receiving more attention as concerns about the future of our planet increase, and it is a resource whose utilization could make an important contribution to establishing a more sustainable living environment [1]. Global industrialization implies an increase in the populations living in small areas, with densely populated centers that present a threat to environmental safety and sustainability, as well food production. The results of the mentioned socio-agriculture management have resulted in global warming and the buildup of chemical and biological contaminants [2]. On the one side is waste material and sustainable agriculture, and on the other is the socio-economic circumstances of living [3]. From this perspective, agricultural wastes containing cellulose are misplaced resources that could be reused in various fields for both environmental and economic benefits. The mediating factor that could enable the transition from harmful waste material into a useful and environmental friendly product is superabsorbent polymers with applications in agriculture. Such polymers present a very desirable class of materials that can be used for different purposes. They have attracted the interest of researchers in many different laboratories. Products made with them fulfil not only their task requirements but also degrade easily in soil [4].

Cellulose is the most common substance in agricultural waste [5]. Moreover, the most abundant renewable carbon source from plant material on Earth is cellulose [6]. It can be extracted from waste materials through chemical processes and be used as a base component for carboxymethyl cellulose synthesis afterwards. In recent years, technological engineers and other scientists have developed new processes in order to produce superabsorbent hydrogels from different waste materials [7]. It was found that chemical methods are more cost-effective in comparison to physical methods [8]. Compared to other natural sources, such as starch and chitosan, cellulose is more readily available [9]. Along with its high absorption properties [10] and biodegradability [11,12], it is desirable and interesting for researchers.

Since not all countries experience sufficient precipitation for agricultural purposes during different times of the year, having a product that allows the slow release of water when it is needed would be very useful. As they can influence the soil permeability and the evaporation and infiltration rates of water through the soils, hydrogels composed of polymers are gaining popularity in agricultural science [13]. 

There are two major classes of hydrogels used in agriculture: (1) those that improve the water supply in soil for plants and (2) those that enable the controlled release of agrochemicals [14]. The affinity for water is adjustable via control of the crosslinking density of matrices [15]. A molecular crosslinked organization stops the burst release of water [14]. The continuous release of water is a property that has received increased interest in recent years. The superb hydrophilic properties and high swelling ratio of hydrogels have led to their recommendation for application in agriculture. CMC is a biocompatible and biodegradable polymer [16].

The aim of this review paper is to change the view of agricultural wastes as a material to be discard to that of a valuable resource that can be chemically modified and find secondary usage in agriculture. CMC is a substance that can be produced by the chemical modification of cellulose from agricultural wastes and afterwards find implementation in the preparation of a biodegradable superabsorbent hydrogel; for this reason, it is highlighted in this paper. 

## 2. Materials and Methods

A detailed review of the available published scientific papers was conducted. Using the Google Scholar database, Scopus, Web of Science and PubMed, electronic research of published papers was performed for the period up to July 2021. There was no exclusion of any language when searching the literature. The used search terms were: cellulose, carboxymethyl cellulose, waste material, superabsorbent hydrogel carboxymethyl cellulose, superabsorbent polymer. We considered whether studies pertained to agriculture, polymer synthesis, sustainable development or biodegradation.

## 3. Useful Waste Material

Millions of tons of agricultural wastes (~731 rice straw, ~354 wheat straw, ~203 corn straw, ~180 bagasse) generated each year present a serious challenge for sustainability [8]. In reality, this waste material is burned [17], generating huge amounts of harmful gases, such as CO_2_, N_2_O and CH_4_; air pollutants, such as CO, NH_3_, NO_x_, SO_2_ and other volatile organic chemicals; and contaminating particulate matter [18]. As the concerns about CO_2_ emissions grow, interest in the utilization of lignocellulosic materials from wastes as abundant sources of substances is growing. This is where the sustainable management of agricultural waste is urgently needed. A move towards green material solutions is a step forward according to a sustainable development policy and environmental neutrality [19,20]. The most important advantage in the use of these materials is that they are affordable worldwide [21].

### 3.1. Characteristics of Cellulose in Agricultural Wastes

Plant biomass is a useful industrial material for researchers focusing on environmental concerns. It consists of cellulose, hemicelluloses, lignin, pectins, gums, mucilages, starch and proteins. Approximately 33% of all plant biomass contains cellulose as the major component because it is the constituent of the rigid cell walls. In fact, cellulose is the most common organic compound on Earth. For example, cotton consists of 90% cellulose and wood contains 40–50%. For industrial use, wood pulp and cotton are the primary sources of cellulose. 

**Figure 1:**
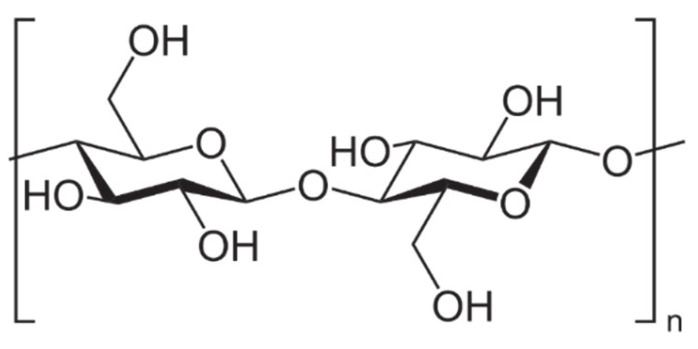
Cellulose chain repeating unit.

The cell walls of lignocellulosic agricultural wastes consist of cellulose (35–65%), hemicellulose (20–45%) and lignin (10–25%). Cellulose (Figure 1) is always the main component, regardless of the content of these three types of biopolymers, which can vary among different plant species [22,23]. In any case, cellulose is chemically described as a linear homopolysaccharide of β-D-glucopyranose units, i.e., a repeating unit known as cellubiose (a dimer of glucose) lined up by β-1,4-linkages [24]. The glucose units in the same chain and between different chains are linked by strong hydrogen bonds due to the hydroxyl groups of the anhydroglucose unit (the monomer of cellubiose) [25]. Because of the strong hydrogen bonding, cellulose is durable and insoluble in water and most organic solvents. The process of removing the non-cellulosic components by different mechanical operations, chemical reactions or biological processes constitutes the extraction process of cellulose from agricultural wastes [8]. 

### 3.2. Waste Material Treatment

A combination of acid–chlorite and alkaline processing is the most commonly used pretreatment method. With the chemical reaction using acid–chlorite, known as delignification or bleaching, most of the lignin and other components is eliminated with a mixture of water, acetic acid and sodium chlorite. The mixture solution with waste material needs to be stirred at 70–80 °C for 4–12 h, and after this process, the solid products of the chemical reaction (holocellulose, consisting dominantly of cellulose and hemicellulose) are washed wish distilled water until pH = 7 and then dried in oven at 50 °C. Then, the solid products need to be treated with an alkaline solution (4–20% (*v*/*v*) sodium hydroxide) for 1–5 h in order to remove the amorphous polymer of hemicellulose and residual lignin. The final solid products obtained after washing with distilled water until pH = 7 and dried in an oven at 50 °C consist mostly of cellulose [24]. 

## 4. Chemical Modification of Cellulose

### 4.1. Carboxymethylation by the Classic Method

Cellulose is an easy target for chemical derivatization and, for this reason, it is used as a raw material in the chemical industry [26]. The carboxymethylation reaction is a multivariable-dependent transformation whose outcome is a waterswellable or water-soluble polymer with highly desirable features [27,28,29,30]. Revised conditions and reagents for the chemical reaction are given in Table 1.

By this method, used by Moussa et al. for the synthesis of CMC from agricultural waste consisting of almond shells, synthesis is carried out in two steps: alkalization of cellulose and its etherification (further details of this chemical reaction will be discussed below). In the mentioned study, during the alkalization of cellulose, 10 g of cellulose was suspended in a solvent solution under magnetic stirring at 80 °C. A volume of 60 mL of NaOH from a 40% aqueous NaOH solution was added to the reaction mixture after 1 h. In order to convert hydroxyl to alcoholate groups, stirring was continued for a further 14 h at the same temperature. Then, the reaction mixture was ready for the etherification reaction, which was conducted by adding 17.4 g of monochloroacetate (MCA). It was stirred for 8 h at a temperature of 80 °C. The etherification process took place as in Equation (2) to obtain CMC and a side reaction that results in the formation of sodium glycolate, as expressed in Equation (3). The surplus alkali was neutralized with 90% (*v*/*v*) acetic acid, and 500 mL of ethanol was added to induce precipitation. The obtained CMC was recovered by filtration and washed with a mixture of ethanol/water 80/20 (*v*/*v*). The final product was washed with pure ethanol and dried in oven at 50 °C for 24 h [31]. 

### 4.2. Microwave-Assisted Synthesis of CMC

By this method, used by Moussa et al. for the synthesis of CMC from agricultural waste consisting of almond shells, almond stems and fig stems, cellulose (3 g) was mixed with 30 mL of water in the reaction vessel. Magnetic stirring at room temperature was performed for 20 min, 20 mL of 40% (*v*/*v*) sodium hydroxide was added and the reaction was performed in a microwave reactor. After this, 5.8 g of MCA was added to the reaction mixture, in order to perform the etherification reaction. This was followed by the neutralization of surplus alkali with 90% (*v*/*v*) acetic acid. For precipitation, 200 mL of ethanol was added. The CMC was collected as a filtrate and washed with a mixture of ethanol/water 80/20 (*v*/*v*) in order to remove salt impurities. The final product was washed with pure ethanol and dried in an oven at 50 °C for 24 h [31].

### 4.3. Proof of Carboxymethylation

#### 4.3.1. Degree of Substitution

After the carboxymethylation of cellulose is achieved, the success and efficiency of the process should be measured. The degree of substitution (DS) of CMC is defined as the average number of hydroxyl groups in the cellulose structure, which was substituted by carboxymethyl and sodium carboxymethyl groups at C2, 3 and 6. 

The degree of substitution is dependent on the reaction conditions for the water-soluble products obtained by carboxymethylation [37]. The variables affecting the DS of CMC are: the concentration of monochloroacetic acid, the temperature and reaction time, the reaction medium and the concentration of NaOH, where a higher number for DS indicates higher solubility of the CMC [52,53,54].

The first chemical reaction is between the hydroxyl groups of cellulose and NaOH, where a more reactive alkaline form of cellulose is generated (1).

CLL–OH + NaOH → CLL–ONa + H_2_O (1)

(Cellulose)(Reactive alkaline form)

This reaction is followed by the reaction of etherification, where CMC is obtained (2), and a reaction in which sodium glycolate is produced as a byproduct (3).

CLL–ONa + Cl–CH_2_–COONa → CLL–O–CH_2_–COONa + NaCl + H_2_O (2)

(CMC)NaOH + Cl–CH_2_–COONa → HO–CH_2_–COONa + NaCl (3)

(Sodium glycolate)

Since the transformation of cellulose to the desired carboxymethyl cellulose takes place during this step, it is of importance to know how each parameter contributes to the product of the chemical reaction. Pushpamalar et al. optimized the reaction conditions for preparing carboxymethyl cellulose from sago waste [49]. They explained the effect of varying parameters in this reaction, including different solvent media, time, temperature, the amount of sodium monochloroacetate and the concentration of sodium hydroxide. 

The effects of different solvent media, such as water, dimethylformamide, methanol, dimethyl sulfoxide, isopropyl acohol, ethanol and butanol, were assessed by Pushpamalar et al. A maximum DS was obtained with isopropyl alcohol as a solvent medium. It was explained that solvent polarities and stereochemistry play a crucial role in this result, and as the polarity of the solvent decreases, the reaction efficiency increases. Regarding the reaction time, there was an increase in DS up to 180 min. By that time, the better reaction environment and prolonged time contributed to the higher availability of molecules for carboxymethylation, favoring the diffusion and absorption of reactants. After 180 min, a decrease was noticed, which may be attributed to the degradation of the polymer. Regarding the obtained results for DS with temperature as a changing parameter, there was an increase up to 45 °C, followed by a decrease. The explanation for this decrease is the degradation of cellulose, or, more precisely, the elimination of molecules of water with the formation of unsaturation on C2, C3 or a ketone functional group on C2. In parallel with this reaction, intermolecular elimination between nearby hydroxyl groups raises the crosslinking by ether linking, decreasing the number of hydroxyl groups available for carboxymethylation. The amount of chemical reactant NaMCA directly affects the formation of NaCMC. An increase in DS occurs until one point, after which it decreases. This is because of the higher concentration of acetate ions. Above the concentration that provides the maximum DS, it is to be expected that sodium glycolate formation is favored. The concentration of sodium hydroxide as a parameter that affects the formation of NaCMC also has one point until DS increases and reaches its maximum. The explanation for this can be found in the carboxymethylation process, where two competitive reactions take place. From the first one, CMC is obtained as a product (Equation (2)), and sodium glycolate is obtained from the second (Equation (3)). For concentrations until the maximum of DS is reached, the first reaction overcomes the second, but above this point, the formation of sodium glycolate prevails due to the inactivation of MCA and its depletion in the side reaction [49].

For the determination of DS, two methods are mostly applied: the USPXXXII method described for croscarmellose sodium, which is practically potentiometric back-titration [41,55], and the “ashing method” [56].

It should be noted that only for Mimosa pigra peel, DS is <0.4 (0.23; Table 1) and thus it could not be used for further investigation in this work.

#### 4.3.2. Infrared Spectroscopy

The instrumental method of choice for confirmation of the carboxymethylation process is infrared spectroscopy. An infrared spectrophotometer allows observation of the carboxymethyl groups on molecules of cellulose. It is used in the process of the chemical transformation of cellulose from *Mimosa pigra* [41]. The instrument registered the same functional groups from cellulose and CMC, such as –OH, –CH_2_, C=O and –O–. However, the difference was noticeable in the high increase in –CH_2_, C=O and –O–, while the band of –OH decreased from the CMC sample when compared with molecules of cellulose itself.

## 5. CMC

**Figure 2:**
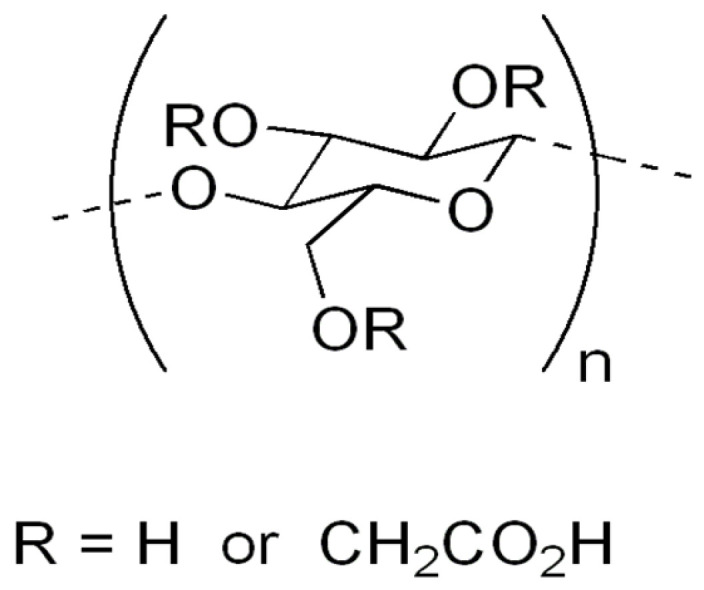
Carboxymethyl cellulose.

Carboxymethyl cellulose (CMC) (Figure 2) is probably the best-known cellulose derivative, which is water-soluble cellulose ether, obtained by reacting sodium monochloroacetate with cellulose in an alkaline medium [9]. This cellulose derivative is an anionic, linear, highly viscous polysaccharide, confirmed as a nontoxic, non-allergenic and biodegradable substance [57,58]. Because all reactions for synthesis are operated at atmospheric pressure, by using commercially available reagents, the production of CMC is simpler than that of most other cellulose ethers [59]. It has a huge range of different applications in different products and processes, such as oil drilling [60], detergents, food exploration, paper, textiles, pharmaceuticals [61] and the agricultural industry [13]. Recently, CMC has found new interest in advanced fields such as tissue engineering [62], dye and lithium ion batteries [63] and the adsorption of heavy metals [64,65,66]. 

This very important and frequently used biopolymer in industry (industrial production of CMC is approximately 360,000 metric tons per year) has partial substitution of the 2, 3 and 6 hydroxyl groups of cellulose by carboxymethyl groups [67,68]. Because of the countless hydroxyl and carboxylic groups provided, it is able to absorb and hold water. The cheapness and simplicity of the production process of this anionic and water-soluble natural polymer has already been shown [49,69,70,71]. The hydrophilicity of CMC increases with an increase in the DS number. Those with DS < 0.4 can swell but remain insoluble, while those with DS > 0.4 are water-soluble and have high viscosity [72]. Therefore, DS is a parameter that directs the final application [29] and thus makes it possible for the material to be applied in agriculture.

### 5.1. Cellulosic Water

A combination of carboxymethyl cellulose, as a cellulosic substrate, and hydrated metallic salts results in the formation of a soft gel named cellulosic water (CW) [73]. In direct physical contact with the growing medium, water is released due to the activity of cellulolytic enzymes of microorganisms living in the growing medium because the gel structure breaks down [57]. This is the mechanism by which soil obtains extra water moisture. CW does not have the ability to become rehydrated and it is applied fully hydrated, with limited contact with the growing medium. Stamps and Savage tested the possibility of delayed wilt of containerized poinsettias held under simulated conditions. It was effective in delaying wilt for 4 weeks [13]. In addition, another study considered the usage of CW for a containerized plant of Rhododendron indicum under greenhouse conditions. The growing medium was supplemented with CW mass 0.086 and 0.172 g/cm^3^ of growing medium. In this way, plants were kept from wilting for 4 weeks [74].

### 5.2. Superabsorbent Hydrogels

Superabsorbent hydrogels consist of three-dimensional networks structured by hydrophilic polymers formed by crosslinking, and they can absorb and hold significant amounts of water [75,76]. When applied on terrain, a superabsorbent hydrogel acts as a water reservoir. As a result, water-depleted regions can overcome hot periods with insufficient rainfall. 

The interest in the application of superabsorbent hydrogels in agriculture is growing [77]. Carboxymethyl cellulose is an essential substance for preparing biodegradable superabsorbent hydrogels for agricultural purposes. This is supported by results that have shown that environmental sustainability and the circular economy in agriculture are promoted by the development of agricultural-waste-derived superabsorbent hydrogels [8]. In Table 2, the chemical composition of superabsorbent hydrogels containing CMC is given.

Due to the simplicity of the composition, the most interesting superabsorbent may be the one consisting only of CMC. The water uptake for 1 g of 10% CMC gel was reported to be ~450 g of water. Swelling behavior is described by second-order kinetics. This material showed better swelling ability compared to hydroxylethyl cellulose and methyl cellulose [79]. The same authors produced a superabsorbent hydrogel consisting of CMC and starch in different ratios. The highest water uptake, ~350 g of water per 1 g of gel, was reported to be achieved with a hydrogel containing 70% CMC and 30% starch [80].

However, the results that are the most thematically significant because they are in accordance with the concept of sustainable agriculture are those obtained by Dai and Huang, who prepared a superabsorbent hydrogel consisting of CMC obtained from pineapple peel, acrylic acid and acrylamide [1], with water uptake of 420.17 g of water per 1 g of superabsorbent; meanwhile, the formulation prepared by Salleh et al. consisted of CMC obtained from oil palm, empty fruit bunches, cellulose and ammonium persulfate and N,N′-methylenebisacrylamide as crosslinkers, which was able to uptake 80 g of water per 1 g of superabsorbent [86].

### 5.3. Enzymatic Degradation of CMC Hydrogel

The term biodegradation has attracted increased attention as plastic materials used in everyday life contaminate the environment. The application of biodegradable polymers addresses this concern because of their conversion into carbon dioxide, water and energy. This desirable feature is limited to aliphatic polyesters, polyethers and some polysaccharides, including cellulose derivatives. Glycosidic linkages bonding polysaccharide rings are targets for microorganisms and hydrolytic enzymes in order to be biodegraded. Cellulose ethers, such as carboxymethyl cellulose, display this feature [90,91,92]. Compared to acrylate- and acrylamide-based hydrogels, these are biodegradable and biocompatible [93].

## 6. Conclusions

Plant waste material is a valuable source of cellulose. It is possible to produce superabsorbent hydrogels from plant waste material via chemical modification, which improves agriculture production. Carboxymethyl cellulose is a substance with a huge range of application, and there are more to be discovered. The discussion presented in this work is relevant to people and industries within different professional fields and has the goal of connecting them in order to achieved sustainable food production and environmental protection. With the ease of implementation, all parties will benefit. It is in everyone’s interest to apply this concept further within society. 

## Figures and Tables

**Table 1:** Reaction conditions for carboxymethylation.

Starting Waste Material	Cellulose (g)	Reaction Medium	NaOH	MCA/ Diazomethane	Temperature (°C)	Reaction Time (h)	DS	Yield (%)	Reference
Almond shells	10	Pentan-1-ol 60 mL	40%, 60 mL	MCA, 17.4 g	80	8	1.72	58.65	[31]
Almond stems	10	Pentan-1-ol 60 mL	40%, 60 mL	MCA, 17.4 g	80	8	2.53	62.77	[31]
Asparagus officinalis stalk end	15	IPA, 350 mL	30%, 50 mL	MCA, 18 g	55	3	0.98	44.04	[32]
Black tea leaves	5	IPA, 80 mL	20%, 10 mL	MCA, 3 g	60	3	0.77	91.2	[33]
Barley straw	1	IPA 10 mL + H_2_O 1 mL	6 M, 5 mL	MCA, 2.4 g	50	3	1.1	106	[34]
Cavendish banana pseudo steam	5	IPA, 100 mL	15%, 20 mL	MCA, 6 g	55	3	0.75	98.63	[35]
Cashew tree gum	5	H_2_O, 5 mL	10 M, 16 mL	MCA, 7.87 g	55	3	2.21	31	[36]
Coconut fibers	5	IPA, 80 mL	20%, 10 mL	MCA, 3 g	50	3	0.65	104.8	[33]
Corn cob	5	IPA, 90 mL	25%, 3.25 mol/AGU	MCA, 2.4 mol/AGU	60	3	1.18	n. r.	[37]
Cotton gin	5	H_2_O:IPA (1:4), 300 mL	20%, 30 mL	MCA, 5 g	40-60	3	0.87	143.7	[38]
Cotton linters	5	H_2_O:EtOH (1:4), 32.8 mL	100%, 7.2 mL	MCA 80%, 10 mL	50	6	0.89	-	[39]
Dried duckweed	5	IPA, 80 mL	20%, 10 mL	MCA, 3 g	60	3	0.54	91.4	[33]
Durian rind fruit	15	IPA, 450 mL	30%, 50 mL	MCA, 18 g	55	3	0.87	165	[40]
Fig stems	10	Butanol, 60 mL	40%, 60 mL	MCA, 17.4 g	80	8	2.83	80.54	[31]
Mimosa pigra peel	10	IPA, 500 mL	50%, 100 mL	MCA, 15 g	55	3.5	0.23	n. r.	[41]
Mulberry paper	15	IPA, 750 mL	40%, 150 mL	MCA, 22.5 g	55	3.5	0.45	n. r.	[42]
Oil palm fibers	5	IPA, 80 mL	20%, 10 mL	MCA, 5 g	50	3	0.6	116.8	[33]
Orange peel	2	IPA, 100 mL	30%, 20 mL	MCA, 3 g	70	6	0.67	n. r.	[43]
Palm bunch	15	IPA, 450 mL	40%, 50 mL	MCA, 18 g	55	3.5	0.36	135.8	[44]
Palm kernel cake	5	IPA, 80 mL	20%, 10 mL	MCA, 3 g	50	3	0.73	116.6	[33]
Papaya peel	15	IPA, 450 mL	40%, 50 mL	MCA, 18 g	55	3.5	n. r.	126.4	[45]
Paper cups	n. r.	EtOH 85%, 150 mL	6 g	MCA, 7 g	70	1.5	1.21	n. r.	[46]
Pineapple peel	10	90% IPA 200 mL + 30% H_2_O_2_ 1.2 mL	50%, 16 mL	Diazomethane 50% (*w*/*v*), 14 mL	45-75	3	1.05	n. r.	[47]
Rice hull	1	IPA 10 mL + H_2_O 1 mL	6 M, 5 mL	MCA, 2.4	50	3	1.3	85	[34]
Rice stubble	5	IPA, 100 mL	30%, 25 mL	MCA, 7 g	50	3	0.64	95	[48]
Sago pulp	5	IPA, 100 mL	30%, 10 mL	MCA, 6g	45	3	0.82	n. r.	[49]
Sugarcane bagasse	5	IPA, 80 mL	20%, 10 mL	MCA, 5 g	50	3	0.69	117.4	[33]
Sugarcane bagasse	5	IPA, 150 mL	25%, 15 mL	MCA, 6 g	60	3.5	0.69	166.2	[50]
Sugarcane bagasse	15	IPA, 450 mL	40%, 50 mL	MCA, 18 g	55	3.5	0.53	142.4	[44]
Sugar beet pulp	2	IPA, 100 mL	30%, 20 mL	3	70	6	0.67	64.2	[51]
Sugarcane straw	5	EtOH 92.5 g + H_2_O 3.7 g	38.46%, 13 mL	MCA, 24.94	60	3	0.4	n. r.	[17]
Wheat straw	1	IPA 10 mL + H_2_O 1 mL	6 M, 5 mL	MCA, 2.4	50	30	1.5	106	[34]

“n. r.”—not reported; “AGU”—anhydrous glucose unit.

**Table 2:** Superabsorbent hydrogels containing carboxymethyl cellulose in their composition.

Composition	Polymerization Method	Swelling Ratio (g/g)	Reference
CMC, acrylic acid, acryl amide, 2-acrylamido-2-methyl-1-propanesulfonic acid, montmorillonite, K_2_S_2_O_8_ (initiator), N,N′-methylenebisacrylamide (crosslinker)	Graft copolymerization	680.2	[9]
CMC, cellulose, epichlorohydrin (crosslinker)	NaOH and urea water solution	1000	[78]
CMC, acrylic acid, acrylamide, crosslinkers (ammonium persulfate, N,N′–methylenebisacrylamide)	Graft copolymerization	420.17	[47]
CMC	Gamma beam irradiation	~450	[79]
CMC, starch	Gamma beam irradiation	350	[80]
CMC, acryl amide, N,N′–methylenebisacrylamide (crosslinker)	Electron beam irradiation	2611	[77]
CMC, acrylic acid, K_2_S_2_O_8_ (initiator), N,N′–methylenebisacrylamide (crosslinker)	Graft copolymerization, inverse suspension polymerization	544.95	[81]
CMC, starch aldehydes, citric acid (crosslinker)	n. r.	87	[82]
CMC, starch, aluminum sulfate octadecahydrate (crosslinker)	n. r.	72	[83]
CMC, cellulose nanofibers, cellulose nanocrystals, citric acid (crosslinker)	Hydroxyethyle cellulose solution	200	[84]
CMC, polyvinylpyrrolidone (crosslinker)	Gamma beam irradiation	144	[85]
CMC, cellulose, epichlorohydrin (crosslinker)	NaOH/urea (7:12) water solution	80	[86]
CMC, acrylic acid, acrylamide, K_2_S_2_O_8_ (initiator), Na_2_S_2_O_5_ (initiator), N,N′–methylenebisacrylamide (crosslinker)	Free-radical grafting solution polymerization	920	[87]
CMC, acrylic acid, KOH (neutralizing agent),	Gamma beam irradiation	~16,500	[88]
CMC, chitin, epichlorohydrin (crosslinker)	NaOH 8% and urea 4% water solution	1200	[89]

“n. r.”—not reported.
